# Within-group behavioural consequences of between-group conflict: a prospective review

**DOI:** 10.1098/rspb.2016.1567

**Published:** 2016-11-30

**Authors:** Andrew N. Radford, Bonaventura Majolo, Filippo Aureli

**Affiliations:** 1 School of Biological Sciences, University of Bristol, 24 Tyndall Avenue, Bristol BS8 1TQ, UK; 2 School of Psychology, University of Lincoln, Brayford Pool, Lincoln LN6 7TS, UK; 3 Instituto de Neuroetologia, Universidad Veracruzana, Xalapa 91000 Veracruz, Mexico; 4 Research Centre in Evolutionary Anthropology and Palaeoecology, Liverpool John Moore University, Liverpool L3 3AF, UK

**Keywords:** conflict, social evolution, behavioural consequences, group living, aggression

## Abstract

Conflict is rife in group-living species and exerts a powerful selective force. Group members face a variety of threats from extra-group conspecifics, from individuals looking for reproductive opportunities to rival groups seeking resources. Theory predicts that such between-group conflict should influence within-group behaviour. However, compared with the extensive literature on the consequences of within-group conflict, relatively little research has considered the behavioural impacts of between-group conflict. We give an overview of why between-group conflict is expected to influence subsequent behaviour among group members. We then use what is known about the consequences of within-group conflict to generate testable predictions about how between-group conflict might affect within-group behaviour in the aftermath. We consider the types of behaviour that could change and how the role of different group members in the conflict can exert an influence. Furthermore, we discuss how conflict characteristics and outcome, group size, social structure and within-group relationship quality might modulate post-conflict behavioural changes. Finally, we propose the need for consistent definitions, a broader range of examined behaviours and taxa, individual-focused data collection, complementary observational and experimental approaches, and a consideration of lasting effects if we are to understand fully the significant influence of between-group conflict on social behaviour.

## 1. Introduction

From ants to humans, conspecific groups form for a variety of reasons that provide benefits to the individuals involved [1]. However, conflicts of interest are also rife in group-living species [2]. Group members can disagree about access to mates or food, the direction of travel or the sharing of tasks [3–5], while individuals in different groups may disagree over possession of territories and their contents [6–8]. Many disagreements are prevented from escalating into aggression by a range of conflict-management strategies (definitions of key terms in the electronic supplementary material, table S1), such as mutual avoidance, signalling or dominance relationships [9–11]. However, when aggressive conflicts (hereafter conflicts) between individuals or groups do arise, they not only carry the risk of injury or death, but can lead to increased anxiety, disrupted social relationships, and alterations in group composition or structure [12–14]. Conflicts thus have the potential to exert a strong influence on subsequent behaviour between group members.

Post-conflict behaviour has been extensively studied in the context of within-group conflict [13,15,16]. Similar changes in behaviour between group members have been found in a variety of taxa, despite considerable interspecific differences in cognitive complexity, diet and phylogenetic history [9,17,18]. Within-group behaviour is also predicted to be influenced by between-group conflict [19,20]. There is strong empirical evidence that the behaviour of human group members towards one another is indeed affected by conflicts with rival groups [21,22], but these studies have considered situations when the out-group threat is still present. Recent work on non-human animals has indicated that behavioural changes can also occur once the immediate threat has passed [14,23–26]. However, while we may expect between-group conflict to have comparable effects on within-group social behaviour across taxa, research on this topic has been restricted to a small number of species. Moreover, behavioural changes in the aftermath of within-group conflict can differ depending on factors like the individual characteristics of participants, and the intensity and outcome of aggression [27,28]. Yet variation in behaviour following between-group conflict has been little studied and thus is poorly understood, despite its likely importance in social evolution.

Our aim is the generation of testable predictions about when and how between-group conflict might shape within-group behaviour in the aftermath. We begin with a brief overview of between-group conflict and then use the extensive literature on the short-term consequences of within-group conflict to provide predictions about how within-group behaviour might be affected following between-group conflict. We consider the types of behaviour that could change and how the role of different group members in the conflict can exert an influence. Furthermore, we discuss how the conflict characteristics and outcome, group size, social structure, and within-group relationship quality might modulate post-conflict behavioural changes. Throughout, we highlight the few empirical studies that have so far tested these predictions. Finally, we discuss the need for consistent definitions, a broader range of examined behaviours and taxa, individual-focused data collection, complementary observational and experimental approaches, and a consideration of lasting effects if we are to understand fully the influence of between-group conflict on social behaviour.

## 2. Between-group conflict

Groups face a variety of potential threats from conspecifics. Individuals, such as immigrant males, may challenge the breeding success of particular group members [25,29]. The presence of an out-group individual may also indicate the imminent attack of another group [30]. Neighbour or unfamiliar groups might attempt to acquire the resources of rivals or annex their territory [6–8]; in these cases, there may be a cost to all or most group members and so a greater incentive for shared defence than when the cost is only to one or a few individuals. The general principles we discuss apply to conspecific out-group threats in general, as it may often be difficult for animals to assess whether they are under threat from one or more individuals, but we mainly focus on conflict between multiple members of different groups for specific examples (see §5 for how the consequences of between-group conflict can differ depending on the type of out-group threat faced). Encounters between groups range from ‘neutral’ interactions, where individuals are in visual or auditory contact and can gather information relating to group composition and breeding opportunities [11], to physical fights that potentially result in injuries or death [31]. Studies on a range of taxa have considered the immediate defensive responses elicited by rival groups and the factors determining the outcome (winning or losing) of interactions with outsiders [7,8,32,33]. However, far less is known about the impacts of between-group conflicts after such interactions have ceased.

Group members often differ in their contributions to between-group conflict due to individual characteristics such as age, sex, kinship and dominance status [6,8,33,34]. Defensive responses may also differ depending on the type of threat; for example, rival groups can be more or less threatening depending on their identity (e.g. neighbour or unfamiliar group), relative size and where they are encountered [10,35,36]. Between-group interactions can themselves vary greatly in characteristics such as duration, intensity and outcome [31,32]. These factors, as well as the availability of group members with whom to interact, within-group social relationships and social structure, are likely to influence post-conflict behaviour.

Compared with research on the behavioural aftermath of within-group conflict, few empirical studies have considered the immediate consequences of between-group conflict [14,23,24,37]. This scarcity is due, at least in part, to the methodological and logistical difficulties that scientists face. For instance, there are generally lower natural rates of between-group encounters than within-group conflicts, and monitoring multiple individuals simultaneously during interactions involving two groups is more challenging [38]. Moreover, whereas it can be feasible to replicate in captivity the conditions required for studying the immediate consequences of within-group conflict [39], doing so for multiple competing groups is difficult, especially for large vertebrates. To provide a predictive framework for the immediate consequences of between-group conflict on within-group behaviour, we therefore draw on the extensive literature investigating how within-group conflict affects subsequent interactions between group members.

## 3. Behavioural responses in the aftermath of conflict

Within-group conflicts are potentially costly, in terms of increased anxiety, the risk of further aggression, and reduced time for feeding or other valuable activities [40,41]. Moreover, conflicts between group members may disrupt social relationships [13,15] and their associated fitness benefits [42]. These social, ecological and emotional costs have selected for conflict-management strategies in the aftermath, such as post-conflict avoidance, submission, aggression and affiliation [13,43]. Between-group conflict is also costly, as it can lead to increased anxiety, resource reallocation, social instability and potential disruption to within-group relationships [14,23]. Thus, post-conflict within-group behavioural changes seen in a within-group conflict context are also predicted following between-group conflicts. To date, between-group conflict studies have focused on post-conflict aggression and affiliation [23,24,37]. We therefore provide detailed predictions relating to these types of behaviour in this section, but emphasize the potential importance of other behaviours in §5.

Within-group conflict can affect the subsequent behaviour of both those individuals involved (combatants) and those that did not participate in the conflict itself (non-combatants) [13,18]. In some species, all group members generally engage actively in conflicts with other groups, albeit with different levels of contribution [6]. In most cases, however, only a subset of the group participates actively in a given between-group conflict [8], although there may be consequences for all group members depending on the outcome. Thus, individuals fulfil one of two main roles: combatants, who were involved in the conflict itself; and non-combatants, who may have observed it or been elsewhere, but did not contribute to the conflict. Because the role of an individual in within-group conflict can influence its subsequent behaviour and interactions with other group members [13,18], predictions about within-group aggression and affiliation following between-group conflict are also likely to depend on whether individuals were combatants or non-combatants.

Post-conflict anxiety (an adaptive response to uncertainty and anticipated threat) can arise either from an individual being involved or viewing a conflict or as a consequence of conflict-induced disruption to within-group relationships [13,44,45]. Although heart rate increases in anxiety-eliciting situations [46], behavioural indicators, such as self-scratching and self-grooming [41,47,48], are more reliable measures of increased anxiety because they have been demonstrated to respond selectively to anxiolytic and anxiogenic drugs [49]. Following between-group conflict, behavioural changes may occur as a by-product of increased anxiety levels, to minimize the negative effects of elevated anxiety in others, or to reduce an individual's own anxiety or that of its group members (predictions 2A–8A, table 1). Anxiety may also underpin other functional explanations for post-conflict changes in behaviour which we discuss in the following subsections.

**Table 1.** Predictions about how between-group conflicts may influence within-group aggressive and affiliative behaviour in the aftermath; predictions are not mutually exclusive.

actor	recipient	prediction	reason	detail
post-conflict aggression				
combatant	combatant	1A	punishment	directed at fellow combatants who did not contribute sufficiently to conflict; most probably dominant individuals targeting more subordinate group members; more likely following lost conflicts
combatant	non-combatant	2A	anxiety	by-product of pent-up anxiety or left-over aggression; more likely following long, high-intensity or lost conflicts; less likely between kin or group members with stronger social relationships
		2B	punishment of free-riders	directed at group members who did not contribute to conflict but who should have done so; most probably dominant individuals targeting more subordinate group members; more likely following lost conflicts; punishment of free-riding may be more likely in smaller groups
		2C	punishment of free-rider's family members	directed at family members of group members who did not contribute to conflict but who should have done so; more likely following lost conflicts; such punishment of free-riding may be more likely in smaller groups
		2D	reducing between-group mating or emigration	herding of relevant group members; most likely to be males herding females, especially when the latter are in oestrous
non-combatant	combatant	3A	reducing receipt of within-group aggression	pre-emptive attacks on returning combatants, especially following long, high-intensity or lost conflicts; likely to be generally rare
non-combatant	non-combatant	4A	anxiety	by-product of general increase in anxiety levels among group members; more likely following long, high-intensity or lost conflicts; less likely between kin or group members with stronger social relationships
		4B	deflection of attention	free-riders attempt to minimize punishment from returning combatants; more likely following lost conflicts
post-conflict affiliation				
combatant	combatant	5A	anxiety reduction	both giving and receiving affiliation can reduce anxiety; could occur between fellow combatants as they are in closest proximity, especially if conflict occurred a long way from rest of group; more likely following long, high-intensity or lost conflicts
		5B	rewarding of contribution	trading of affiliation (e.g. hygienic function of allo-grooming, anxiety reduction) for participation in recent conflict
		5C	signal of group cohesion	directed at rival group as a display of strength to minimize further between-group aggression
combatant	non-combatant	6A	anxiety reduction	directed at any group members, though may be more prevalent between individuals with stronger social relationships; more likely following long, high-intensity or lost conflicts
		6B	increasing future contributions	trading of affiliation for increased participation in future conflicts; directed at group members who should contribute to conflicts; most likely when relative group size influences conflict outcomes
non-combatant	combatant	7A	reducing receipt of within-group aggression	pre-emptive affiliation, especially following long, high-intensity or lost conflicts and by free-riders who would be potential targets for punishment; may be more likely in more despotic species
		7B	rewarding of contribution	trading of affiliation for participation in recent conflict; for instance, females rewarding males in those species in which only the latter engage with rival groups; more likely following conflicts that were won
		7C	consolation	response to returning combatants exhibiting anxiety, especially following long, high-intensity, lost conflicts; particularly likely between group members with stronger social relationships or in kin groups; more likely in less despotic species
non-combatant	non-combatant	8A	anxiety reduction	among individuals witnessing a conflict, especially following long, high-intensity or lost conflicts; may be more prevalent between group members with stronger social relationships
		8B	relationship strengthening	affiliation may strengthen social relationships between free-riders, making them more likely to assist one another in future conflicts

### (a) Post-conflict aggression

Following within-group conflict, further aggression can arise between combatants. This renewed aggression is often explained in terms of winner and loser effects, whereby winning a conflict favours further wins and losing elicits further losses [50]. Renewed aggression can also function to signal the fighting abilities of the aggressor to bystanders and help the former to maintain or raise their dominance rank [51]. Following between-group conflict, there is no direct within-group parallel in terms of renewed aggression, as the former opponents are from a different group. However, subsequent aggression between members of the same group who previously fought alongside one another against another group might also be expected in some situations (prediction 1A, table 1). For instance, post-conflict aggression could be an act of punishment if certain combatants contributed less than expected (acted as free-riders) in the conflict itself [52]. The occurrence of punishment, as with all post-conflict behaviour, is likely to be influenced by other factors (§4); in this case, for example, it may be more prevalent following lost conflicts.

Aggression following within-group conflict can also involve non-combatants, usually bystanders. Recent victims may attack non-combatants to redirect the attention of the original aggressor and others away from themselves and thus reduce the loser effect [53]. Kin-oriented redirected aggression, where combatants are aggressive towards relatives of their opponent, has been observed [54,55]; it may be an act of ‘revenge’ or a means by which recent victims reduce the risk of renewed aggression by the original aggressor [55]. Following between-group conflict, there are no direct within-group parallels in terms of former opponents, as these are from a different group, but combatants might still be aggressive towards non-combatants (predictions 2A–D, table 1). As with combatant–combatant interactions, post-conflict aggression directed towards non-combatants could represent punishment of free-riders. Combatants could also punish family members of the free-riders, in groups where more than one kin unit is present. Aggression by combatants could involve herding behaviour, which may be a means of preventing emigration or mating between animals from different groups [56]; that is, males may herd females to prevent paternity loss. Herding is more likely to occur during the mating season (in seasonally breeding species) or when there are oestrous females in the group, and be directed from males to females (especially in sexually dimorphic species where males are much larger than females).

Aggression following within-group conflict can be initiated by bystanders. If directed towards a previous combatant, bystanders are more likely to attack those who lost the initial conflict [57], as they have more chance of winning against recent losers, and thus of enhancing their dominance rank. In a between-group context, it is possible that non-combatants might pre-emptively attack returning combatants, to reduce the likelihood of punishment directed towards them (prediction 3A, table 1). This is unlikely to be common, otherwise groups might disintegrate as the consequence of escalated aggression in the aftermath of between-group conflict.

Bystander-initiated post-conflict aggression in a within-group conflict context can also be directed towards other bystanders [58]. Individuals may be signalling their fighting ability or attempting to deflect attention and thus minimize the likelihood of redirected aggression from former combatants. Following between-group conflict, non-combatants might also attack one another (predictions 4A–B, table 1) if, for instance, free-riders are trying to deflect attention and avoid punishment from returning combatants.

There is limited empirical evidence to date for an increase in within-group aggression following between-group conflict; this probably reflects a lack of research, rather than a general absence of such behaviour. A study of ring-tailed lemurs (*Lemur catta*) found no such increase in aggression [59]. However, combatants in male bonnet macaque (*Macaca radiata*) between-group conflicts showed more aggression to non-combatant females after than before the conflict [56]. Male aggression targeted at own-group females could function as herding behaviour [56], or could be the consequence of increased anxiety; it is unlikely to be punishment because it is the males in this species who engage in conflicts with rival groups. In tufted capuchins (*Sapajus apella*), while there was no increased within-group aggression in the aftermath of between-group conflict, aggression rates were higher when visual interactions were possible with a rival group compared with when a barrier hid the neighbours from view [24]. These findings support the view that increased anxiety arising from between-group conflict can result in subsequent increases in within-group aggression.

### (b) Post-conflict affiliation

Affiliation between combatants, especially former opponents, occurs often in the aftermath of within-group conflict. The most commonly suggested function is reconciliation [60], with opponents who engage in post-conflict affiliation resuming regular interactions sooner, showing more tolerance towards each other, and being less likely to receive further aggression from each other and bystanders than opponents who do not reconcile [16]. If third parties have supported one or more of the combatants, the latter may offer affiliation as a reward for their contribution [61]. There is no direct equivalent of reconciliation in the context of between-group conflict, as former opponents are in different groups. However, post-conflict affiliation between combatants from the same group is still predicted (predictions 5A–C, table 1). For instance, it might be used to reward individuals for their contribution to the conflict [23] given that affiliation is traded for other commodities in a variety of contexts [62]. Affiliation may also potentially signal group cohesiveness to rivals, which could reduce the likelihood of further conflict with them [63].

Affiliation following within-group conflict can involve non-combatants. Former combatants, especially victims, may seek affiliation with bystanders to lessen the risk of further aggression from previous opponents or of new aggression from bystanders [44,64]. Post-conflict affiliation initiated by the former victim can also serve as reconciliation, aiding the restoration of the relationship between former combatants when the risk of renewed aggression from the former aggressor is too high to reconcile directly [18]. Combatant-initiated affiliation with non-combatants is also predicted to occur following between-group conflicts (predictions 6A–B, table 1). For example, former combatants might initiate affiliation with non-combatants, especially free-riders, as a trade for their future contributions to between-group conflicts. This might be particularly important if relative group size influences conflict outcome [32], though may be more likely in advance of a conflict [65], rather than in the aftermath.

Bystander-initiated affiliation with former combatants in the aftermath of within-group conflict can serve a self-protective function, reducing the risk of the bystander, or their kin, receiving redirected aggression [18,45]. Post-conflict bystander-initiated affiliation with a former combatant has also been suggested to substitute or facilitate reconciliation (in terms of restoring baseline tolerance levels between former combatants), when the bystander is kin or has a strong relationship with the other former combatant [18,66], or may calm the recipient and function as consolation [60,67]. In a between-group conflict context, affiliation initiated by non-combatants towards combatants may also be predicted (predictions 7A–C, table 1). Bystander-initiated affiliation may reduce the risk of redirection, and may be particularly beneficial if bystanders are free-riders and thus at risk of punishment from combatants [68]. Alternatively, bystanders may initiate affiliation with combatants as a form of ‘payment’ for the benefits gained from successful defence of resources and protection from intruders. In principle, bystanders may initiate affiliation as a form of consolation to combatants who have lost, at least in those species where consolation is deemed plausible.

Post-conflict affiliation between bystanders has been demonstrated in a small number of studies on within-group conflict [58,69]. Bystanders affiliate preferentially with group members with whom they have a strong social relationship [69]; such affiliation likely reduces their anxiety [70]. Non-combatants witnessing a between-group conflict might similarly be predicted to affiliate with one another in the aftermath (prediction 8A, table 1). Such bystander–bystander affiliation may serve to strengthen relationships between group members (prediction 8B, table 1), which in turn might reduce the risk of free-riding during future conflicts if individuals are more likely to assist those with whom they have strong relationships [71].

There is some empirical evidence for changes in within-group affiliation following between-group conflict. While a post-conflict change in affiliative behaviour was not found in ring-tailed lemurs [59] and vervet monkeys (*Cercopithecus aethiops* [72]), blue monkeys (*C. mitis*) and samango monkeys (*C. mitis erythrarchus*) increased allo-grooming of group members in the aftermath of between-group conflicts [63,73]. No detailed data are available on partner choices or how the grooming relates to participation in the preceding conflict, so conclusions about the function are speculative. Affiliation may potentially signal group cohesiveness to rivals, which could reduce the likelihood of further conflict with them [63]. Female bonnet macaques groomed and mated with males that had participated more in recent between-group conflict [56], which suggests that they might have been rewarding combatants. Experimental manipulations inducing aggressive interactions between focal groups and single out-group individuals led to post-conflict increases in within-group affiliation in cooperatively breeding cichlid fish (*Neolamprologus pulcher* [25]) and Wied's black tufted-ear marmosets (*Callithrix kuhli* [74]); in the latter study, there was a greater increase in larger compared with smaller groups. Post-conflict within-group allo-preening increased in the green woodhoopoe (*Phoeniculus purpureus*), a cooperatively breeding bird in which all group members participate in between-group conflicts and are thus combatants [23,37]. Increased preening of subordinate groupmates by dominants suggested the former were being rewarded for their contribution. In this species, relative group size is important in deciding the outcome of between-group conflict [32] and subordinates contribute more than dominants to such interactions [6].

## 4. Factors modulating post-conflict behaviour

The type and frequency of behavioural responses in the aftermath of a conflict is likely to be modulated by a number of factors that can affect our predictions (table 1).

### (a) Conflict characteristics

The duration and intensity of within-group conflicts can affect post-conflict behaviour [16]. For instance, if longer and more intense interactions increase anxiety levels more than shorter, less intense ones, then the former can have a greater impact on post-conflict aggressive and affiliative behaviour. The characteristics of between-group conflicts are similarly expected to influence within-group behaviour in the aftermath (predictions 2–8A, table 1). The duration of interactions between green woodhoopoe groups is positively correlated with the rate of post-conflict allo-preening among group members [23]. As rival group identity (e.g. neighbour versus unfamiliar) affects perceived threat levels and thus conflict intensity in a variety of species (e.g. [10,35]), it too should influence post-conflict within-group behaviour. A playback experiment on green woodhoopoes demonstrated a greater increase in within-group allo-preening following simulated territorial intrusions by unfamiliar groups compared to neighbours [37]; while neighbours probably only intrude temporarily, unfamiliar groups may usurp residents permanently. By contrast, there was a greater increase in post-conflict affiliation by *N. pulcher* (cichlid fish) group members following simulated intrusions by neighbours than strangers [25]; in this species, neighbouring individuals are more likely than unfamiliar individuals to take over breeding or dominance positions.

### (b) Conflict outcome

Losing a within-group conflict probably results in greater anxiety than winning, either because losing is inherently more stressful or because there is a greater risk of victims receiving further aggression than their former opponent [66,67]. Consequently, losers of within-group conflict often initiate more affiliation with bystanders, and receive more from them, than do winners [44,75]. The outcome of between-group conflicts is also expected to influence within-group post-conflict behaviour for similar reasons [23]. In green woodhoopoes, an increase in post-conflict allo-preening was most apparent following long conflicts that were lost, and it was driven primarily by the dominant pair preening subordinate group members [23]. In addition to the higher need for anxiety reduction following losses (predictions 5A and 6A, table 1), increased affiliation in the aftermath may enhance relationship strength between individuals, and thus group cohesion, and perhaps increase the likelihood of subordinate help in future conflicts (prediction 6B, table 1). Female white-faced capuchins (*Cebus capucinus*) are more likely to groom the alpha male following between-group conflicts that are won [68]. Such post-conflict behaviour may represent an example of non-combatants rewarding combatants for maintaining a collective resource or protecting them from outsiders (prediction 7B, table 1).

### (c) Group size

Just as group size can potentially influence behavioural interactions following within-group conflict [76], so it may play a role in the aftermath of between-group conflict. For instance, if there are more equitable contributions to conflict by members of smaller groups [77], this could result in a more even spread of post-conflict affiliation. If relative group size affects the outcome of between-group conflicts [32], then biological market dynamics may be important. For example, individuals in smaller groups may have a greater need to ensure future contributions from group members, via increased post-conflict aggression or affiliation (predictions 2B and 6B, table 1). Alternatively, dominants in smaller groups may be less willing to punish free-riders because subordinates are relatively more valuable than those in larger groups [78]. Free-riding may be more likely in larger groups, although it may also be harder to detect, which in turn could reduce the likelihood of post-conflict punishment (predictions 2B and 2C, table 1). In general, the likely greater differences in the roles and contributions of individuals from larger groups during conflicts, and more unequal distribution of the resources at stake (see §5), could result in greater selectivity for targets and partners of post-conflict aggression or affiliation. There could also be indirect effects of group size. For example, there may be greater partner availability for post-conflict interactions in larger groups, potentially resulting in increased levels of affiliation and aggression as has been seen following within-group conflicts [27,28].

### (d) Social structure

The network and strength of social relationships an animal has in their group is a predictor of post-conflict behaviour in a within-group conflict context [79]. Within-group social structure could similarly influence interactions in the aftermath of between-group conflict (predictions 3A, 7A and 7C, table 1). Inter-specific differences in social structure can lead to variation in the risks of collective action problems [80], of which between-group conflict is a classic case, which could in turn affect post-conflict behavioural interactions. For example, contribution to between-group conflict is more equal across group members, and within-group post-conflict affiliation appears stronger, in cooperatively breeding green woodhoopoes [23,37] than in various primate species living in multi-male–multi-female groups [26,72]; in societies where free-riding is more common, pre-emptive appeasement may also be more likely [80]. A major expansion in the number of taxa studied is required to test whether this reflects a more general effect of within-group social structure, including the possibility that animals in groups composed of genetic relatives (e.g. family units in cooperative breeding species) may be more likely to show high levels of post-conflict affiliation than animals from groups where overall genetic similarity among group members is low [23,74]. The broader population social structure (e.g. the number and proximity of neighbouring groups) might also be expected to have an important influence on within-group behaviour, especially in species with high population densities. This could be a direct effect—more neighbours results in more frequent between-group conflict—or an indirect effect if, for instance, post-conflict within-group behaviour is influenced by the presence of an audience [25].

### (e) Within-group relationship quality

Affiliation following within-group conflict can be strongly influenced by the overall quality of the relationship between combatants [13]. The stronger a social relationship, the greater the cost of its disruption [12,41], and thus affiliation is more likely to be observed after conflicts between combatants who have stronger social relationships [28,81]. We predict relationship quality also to modulate between-group post-conflict behaviour: group members having stronger relationships should exchange lower frequencies of post-conflict aggression and higher frequencies of post-conflict affiliation than those having weaker relationships. For instance, it may be less likely that returning combatants take out pent-up anxiety on individuals with whom they have a stronger relationship (prediction 2A, table 1). Similarly, affiliation may be more likely used to reduce the anxiety of group members with whom the giver has a stronger relationship (predictions 6A and 8A, table 1) or to console such individuals (prediction 7C, table 1).

## 5. The future

In addition to the predictions and their modulating factors addressed in the previous sections, five key points need to be considered as research into the consequences of between-group conflict moves forward. First, it would be beneficial to standardize what is defined as the endpoint of a between-group conflict, and thus the time from which post-conflict behaviour is assessed. Between-group conflicts are often considered finished only when the interacting groups move a particular distance apart [34,36] (but see [32]); a thoughtful discussion on this issue is provided in [38]. The exact distance at which a between-group conflict is deemed finished is decided on the basis of such factors as ecology (e.g. habitat density), daily travelled distance and home-range size [8]. By contrast, researchers focusing on within-group conflict usually start post-conflict behavioural assessment immediately after the relevant interactions are terminated [13,27]. We propose that defining between-group conflicts, including their endpoint, on the basis of the temporal occurrence of the relevant aggressive behaviour would allow more meaningful comparisons both between species and between the different types of social conflict (e.g. those arising within and between groups).

Second, our predictions (table 1) relate to post-conflict aggression and affiliation, because these have been the focus of between-group studies to date. However, a broader range of behaviours (e.g. avoidance, submission) are likely to be influenced. For example, submission may be used to reduce conflict-induced anxiety [82]; individuals who participate more in a between-group conflict may show reduced submission in the aftermath if there is less need to appease dominants, while those who contribute less may be more submissive to minimize the risk of punishment. Analysing a wider range of interactions, and comparing how different types of behaviour are affected by the same conflict (e.g. [24,25]), may also help to distinguish between potential functions. For instance, if individuals are seeking to reduce the anxiety of other group members, then post-conflict affiliation may be expected to increase and aggression to decrease; by contrast, when combatants try to ensure future contributions from bystanders, both affiliation and aggression may increase. Not all types of interactions should occur in every species, which is another reason why a wider taxonomic spread is important (see also §4d).

Third, the few studies conducted to date on the consequences of between-group conflict for within-group behaviour have tended to consider the mean responses of all group members [26,72] (but see [23,37]). The often-used spatial definition of the endpoint of between-group conflict (see above) also implicitly assumes that groups act as a cohesive unit where individual contribution to the conflict is qualitatively, quantitatively and temporally coordinated among group members. However, individuals differ in many key characteristics likely to influence post-conflict behaviour, including if, for how long and how they have participated in the conflict (see §§2–4). Because between-group conflicts can last from a few minutes to several hours, an animal could be aggressively involved with another group at some stages, engaged in vocal exchanges at other points, and not be involved at all during the remainder of a particular conflict. Moreover, the assumptions about completely coordinated action between group members are rarely met [8,33], at least partly because the relative threat to different group members is likely to differ depending on the identity of the opponents; for example, whether there are intrusions by single individuals seeking reproductive opportunities or several individuals looking to take-over territory space. Finally, post-conflict interactions may be influenced by the resource at stake and by whether the benefits are likely to be shared between all or most group members or only a few. It is therefore imperative that studies adopt an individual-focused assessment of between-group conflict and subsequent post-conflict behaviour. Such an approach can take into account opponent and conflict characteristics, thus tracking post-conflict behavioural responses of individuals relative to the threat they face and their own contribution to the between-group conflict. This kind of dynamic assessment already occurs in the context of within-group conflict, where post-conflict behavioural recordings are postponed if former opponents exchange further aggression within a defined period of time from the former conflict [76,83].

Fourth, a complementary approach, combining both natural observations and experiments, is likely to be especially important when studying the consequences of between-group conflict. Observations of full interactions between wild groups are paramount, not least to establish baseline levels of conflict and the range of natural behaviours seen both during the interactions (e.g. long-distance calls, visual displays, physical aggression) and in the aftermath. Experiments can subsequently allow controlled consideration of particular aspects of post-conflict behaviour. In captive conditions, there is the possibility to simulate intrusions by movement of rival individuals or groups into established territories [25,77] or by simply providing visual exposure to neighbouring groups [24,72]. In the wild, and in those species identified from natural observations as using them, relevant vocalizations could be played back [10,84] before examining within-group behaviour in the aftermath [37]. Playbacks cannot fully simulate naturally occurring interactions because the level of involvement exhibited by the study individuals, as well as their post-conflict behaviour, depends on the actions of members of the opposing group [38]. Moreover, playback of a single (combined) vocalization from a rival group only replicates the start of what could be an extended exchange of alternating vocalizations between groups [6]; interactive playbacks with the experimenter responding in real time to the vocalizations of the focal group could therefore be beneficial. It is important to point out, however, that great care must be taken with experimental manipulations, given the potentially profound consequences of even simulated between-group conflict; ethical considerations are particularly relevant in this context and should be informed by previous detailed natural observations.

Fifth, our focus has been on how between-group conflict affects within-group interactions in the immediate aftermath. However, the link is almost certainly a dynamic two-way process—within-group behaviour, social structure and relationships are likely to affect participation in between-group conflict [65,85]—and the consequences of between-group conflict may be longer lasting. For instance, between-group conflicts might influence collective decisions relating to resource use over periods of days or weeks [14,86], which in turn could affect population structure by altering the spatial distribution of groups within the habitat (both temporarily and permanently). Consensus decision-making may be more likely if, for example, group cohesion is enhanced by post-conflict increases in affiliation [86]. There is also the potential for between-group conflict to impact individual fitness, not only through immediate direct effects on survival [7,31], but also through changes in space use, resource access, vulnerability to predators, exposure to disease and reproductive success [38]. In the latter case, the stress of territorial intrusions might delay breeding and affect offspring growth, health and survival [87]. Post-conflict behaviours that lessen anxiety may therefore reduce this impact. Finally, between-group conflict could act as a powerful selective force with respect to within-group behaviour more generally, not just in the aftermath of conflict—for instance the levels of affiliation and cooperation shown outside of conflict periods [26]—and social structure, alone or in combination with within-group conflict [3,71,88]. Future studies would therefore benefit from considering not only short-term consequences but also more lasting potential effects of between-group conflict.

## 6. Conclusion

Theory predicts that between-group conflict should influence within-group behaviour, and recent evidence from primates, birds and fish suggests that such a link is likely to be taxonomically widespread. Our aim is to stimulate further empirical research in this field—our knowledge about the influence of between-group conflict lags behind many other aspects of social behaviour—both to build a larger evidence base and to consider more detailed aspects of the relationship between out-group threats and within-group processes. Exploring the similarities and differences between species, and comparing the impacts of within- and between-group conflict, will allow greater understanding about sociality and its evolution and maintenance. In discussing a range of fundamental behavioural issues, such as conflict management, punishment, collective-action problems, anxiety and intra-population behavioural flexibility, we demonstrate that between-group conflict and its consequences pertain to a broad suite of biological research. Moreover, since the management and consequences of conflict are of more general importance, including to human society and global politics, more focused assessment of between-group conflict has relevance for biology, anthropology, economics, psychology, and the social and political sciences.

## Supplementary Material

Electronic Supplementary Material for Radford et al.
