# Supplementary material for: Within-group behavioural consequences of between-group conflict: a prospective review
Source: Proc Biol Sci. 2016 Nov 30;283(1843):20161567. doi: 10.1098/rspb.2016.1567 (PMC5136580; doi:10.1098/rspb.2016.1567)
Supplement: Electronic Supplementary Material for Radford et al. [file rspb20161567supp1.pdf]

Within-Group Behavioural Consequences of Between-Group Conflict: A Prospective Review

Andrew N. Radford, Bonaventura Majolo & Filippo Aureli

Supplementary Material

Supplementary Table 1. Definitions of key terms used in the paper.

Term	Definition
Affiliation	The exchange of 'friendly' behaviour, such as allo-grooming, allo-preening, gentle body or muzzle contact, embraces and vocalisations. Used post-conflict, these behaviours can reduce anxiety, re-establish/strengthen relationships, act as a reward, be used as a trading commodity or help console.
Aggression	Attacks with or without physical contact on other individuals (including herding behaviour). Can refer to initial conflict (within or between groups) and post-conflict behaviour. Post-conflict aggression may be a by-product of increased anxiety or be used to deflect attention, punish free-riders or reduce between-group emigration and mating.
Bystanders	Animals not involved in the conflict (i.e. non-combatants), who have witnessed the conflict.
Combatants	Individuals involved in a conflict. In the context of within-group conflicts, this includes both former opponents and third-party supporters; in the context of between-group conflict, this refers to all group members who interacted aggressively with conspecifics from outside the group. Combatants can subsequently be involved in post-conflict behavioural interactions with other combatants and/or non-combatants.
Conflict	An aggressive interaction between two or more animals. It can occur between two members of the same group (within-group conflict) or between two or more conspecific animals belonging to different groups (between-group conflict), and can be of variable intensity.
Conflict-management strategies	Behaviours that have evolved to minimise the likelihood of within-group or between-group disagreements escalating into conflicts, and behaviours that have evolved to mitigate conflicts in the aftermath.

Consolation	Post-conflict affiliation initiated by bystanders towards former combatants, which reduces the former combatant's post-conflict anxiety.
Former opponent(s)	In the aftermath of a conflict, the individual(s) who has recently exchanged aggression/submission (i.e. former aggressor and former victim); a subset of combatants.
Free-riders	Group members who gain benefits from the actions of others, without paying the costs of being involved themselves.
Herding	Aggressive action (usually by males) that prevents emigration or mating with animals from different groups (by females).
Intensity of conflict	Conflicts vary depending on the type of aggressive pattern displayed (ranging from long-distance threatening displays to physical attacks).
Non-combatants	Individuals not involved in the conflict. May have witnessed the conflict (bystanders) or may have been elsewhere (non-bystanders). They may be involved in post-conflict behavioural interactions with combatants and/or other non-combatants.
Outcome of conflict	A conflict can have a clear-cut result (i.e. one or more winners and one or more losers) or be undecided, depending on whether uni-directional aggressive and submissive behaviours are displayed.
Out-group threat	Threat posed to group members by one or more conspecific individuals from outside the group.
Post-conflict	The aftermath of a conflict where changes in the aggressive or affiliative behaviour can be observed. Traditionally, research on post-conflict behaviour has focused on the first 10–60 min after a conflict.
Redirected aggression	Post-conflict aggression from former victims towards non-combatants. A special case of redirection is kin-oriented redirection (sometimes called 'between-family revenge'), in which a victim is aggressive towards a relative of the former aggressor, to inflict indirect costs to the aggressor and deter them from further aggression.
